# Spatial Pattern Analysis and Conservation Assessment of Apiaceae in Mongolia

**DOI:** 10.3390/plants13182635

**Published:** 2024-09-20

**Authors:** Magsar Urgamal, Shukherdorj Baasanmunkh, Zagarjav Tsegmed, Batlai Oyuntsetseg, Chuluunbat Javzandolgor, Sheng-Xiang Yu, Jung-Won Yoon, Magdalena G. W. Cygan, Hyeok Jae Choi

**Affiliations:** 1 Laboratory of Plant Systematics and Phylogenetic, Botanic Garden and Research Institute, Mongolian Academy of Sciences, Ulaanbaatar 13330, Mongolia; urgamalm@mas.ac.mn (M.U.); javzandolgor@ibcas.ac.cn (C.J.); 2 Department of Biology and Chemistry, Changwon National University, Changwon 51140, Republic of Korea; baasanmunkh.sh@gmail.com (S.B.); tsegmidzagarjaw@gmail.com (Z.T.); 3 Department of Biology, School of Arts and Science, National University of Mongolia, Ulaanbaatar 14201, Mongolia; oyunaa@num.edu.mn; 4 State Key Laboratory of Plant Diversity and Specialty Crops, Institute of Botany, Chinese Academy of Sciences, Beijing 100093, China; yushengxiang@ibcas.ac.cn; 5 University of Chinese Academy of Sciences, Beijing 100049, China; 6 China National Botanical Garden, Beijing 100093, China; 7 DMZ Botanic Garden, Korea National Arboretum, Pocheon 11186, Republic of Korea; kokokoss@korea.kr; 8 International Union for Conservation of Nature, Biodiversity Assessment and Knowledge Team, Cambridge CB2 3QZ, UK; magdalena.cygan@iucn.org

**Keywords:** conservation status, species richness, protected areas, vascular plants, weighted endemism

## Abstract

The family Apiaceae, distributed throughout the Northern Hemisphere, is the largest family of angiosperms. However, little is known about the conservation status, diversity, and distribution of Apiaceae species in Mongolia. This study had two main aims: (1) to assess the national status of Apiaceae species under IUCN Red List Criterion B; (2) to evaluate the species diversity and richness of Apiaceae across Mongolia. We utilized ConR packages to assess the national Red List status of all known Mongolian Apiaceae species by analyzing their most comprehensive occurrence records. The results indicated that 27 species were classified as threatened, including 4 Critically Endangered (CR), 9 Endangered (EN), and 14 Vulnerable (VU) species. Meanwhile, 39 species were assessed as non-threatened, with 2 Near Threatened (NT) species and 37 species of Least Concern (LC). Furthermore, detailed distribution maps for 66 Apiaceae species in Mongolia were presented. We assessed the species diversity and Shannon and Simpson diversity indices of Apiaceae by analyzing all occurrence records using the iNext package. Overall, the Hill diversity estimates indicate that the sampling conducted in Mongolia adequately captured species occurrences. For species pattern analysis, we examined the species richness, weighted endemism, and the corrected weighted endemism index using Biodiverse v.4.1 software. Mongolia was portioned into 715 grid cells based on 0.5° × 0.5° grid sizes (equivalent to approximately 50 × 50 km^2^). There was a total of 3062 unique occurrences of all Apiaceae species across Mongolia. In the species richness analysis, we identified 10 grids that exhibited high species richness (18–29 species) and 36 grids with 11–17 species. For genus richness, we observed seven grids that exhibited a high genus richness of 16–22 genera. Furthermore, we analyzed species richness with a specific focus on threatened species, encompassing CR, EN, and VU species throughout Mongolia. A total of 92 grids contained at least one threatened species. There were six grids that had two to five threatened species, which were adequately covered by protected areas in western Mongolia. Overall, our results on species richness and conservation status will serve as important foundational research for future conservation and land management efforts in Mongolia.

## 1. Introduction

Biodiversity hotspot and gap analyses are standard approaches for identifying priority areas for species conservation. Hotspots are defined as either the most important sites in terms of species diversity or sites where the most threatened or endemic species occur [1]. In recent years, a number of publications have focused on species pattern diversity, using species occurrence records at both global and regional scales [2,3,4]. Additionally, several studies have focused on specific families [5,6] or genera [7,8,9].

Assessing the IUCN Red List status of a species is important for establishing conservation priorities [4,10]. Since 2011, approximately 640 species (representing 21% of the 3041 native taxa based on [11]) of vascular plants have been evaluated using regional red lists in Mongolia [6,12,13,14]. Among these, 390 and 250 species were classified as threatened (Critically Endangered, Endangered, and Vulnerable) and non-threatened (Near Threatened, Least Concern and Data Deficient), respectively. Most of the assessed species have been evaluated by committee members, such as botanists, in Mongolia [12,14]. However, owing to limited data capture and field survey efforts, representative sizes of the extent of occurrence (EOO) and area of occupancy (AOO) of most species remain lacking. For example, the EOO and AOO have been determined for only 5% of Red Listed species using GeoCAT [13] and ConR [6]. Overall, 79% of vascular plants in Mongolia have not been evaluated at the national level.

The family Apiaceae is the largest family of angiosperms, and it is widely distributed in the temperate zones of both the Northern and Southern Hemisphere [15]. It has considerable species diversity, primarily being concentrated in Central Asian countries [16,17,18,19,20]. This family comprises 466 genera and approximately 3800 species, which include many important vegetables and medicinal plants [21,22]. Mongolia is renowned for its diverse landscapes, ranging from the Gobi Desert to mountainous regions, where a variety of plant species have adapted to thrive in different environmental conditions [21]. The flora of Mongolia exhibits remarkable adaptability to the harsh climatic conditions of Mongolia, which are characterized by extreme temperatures, aridity, and high altitudes [22]. To date, a total of 3041 native vascular taxa, belonging to 653 genera and 111 families, have been identified in Mongolia [11]. 

Apiaceae is one of the largest families in the flora of Mongolia [11]. The first comprehensive checklist of Apiaceae was published by Grubov [23] and included 46 species and 26 genera in Mongolia. A taxonomic revision of Apiaceae with identification keys and regional distribution was provided by Urgamal [24]. Recently, Pimenov [18] updated and revised the Chinese Apiaceae, which included and discussed the majority of the Mongolian Apiaceae species. To date, a total of 66 native taxa belonging to 36 genera in the Apiaceae family have been recognized according to the latest checklist of native Mongolian vascular plants [11]. In addition, three non-native Apiaceae species are found in Mongolia: *Anethum graveolens* L., *Eryngium planum* L., and *Pastinaca sativa* L. [11]. Of all the Mongolian Apiaceae, eight species are categorized as subendemic to Mongolia because they co-occur in Russia and China [11]. To date, no Apiaceae species have been identified as endemic to Mongolia [6].

Apiaceae species have previously been assessed at the national level using the International Union for Conservation of Nature (IUCN) Red List Categories and Criteria [12,14]. In addition, the distribution maps of several species have been updated recently [25,26]. However, numerous important species, especially those of medicinal value, have not been evaluated using Red List Categories at the national level. Furthermore, the species diversity and richness of Mongolian Apiaceae have not been evaluated to date because of incomplete species occurrence data. Therefore, our study aimed to (1) analyze Apiaceae species richness using data from all known locations across Mongolia; (2) assess the national IUCN Red List status of all Mongolian Apiaceae species using Criterion B; and (3) determine the diversity of threatened Apiaceae species in Mongolian protected areas.

## 2. Results

### 2.1. Conservation Assessment and Distribution

**Figure 1:**
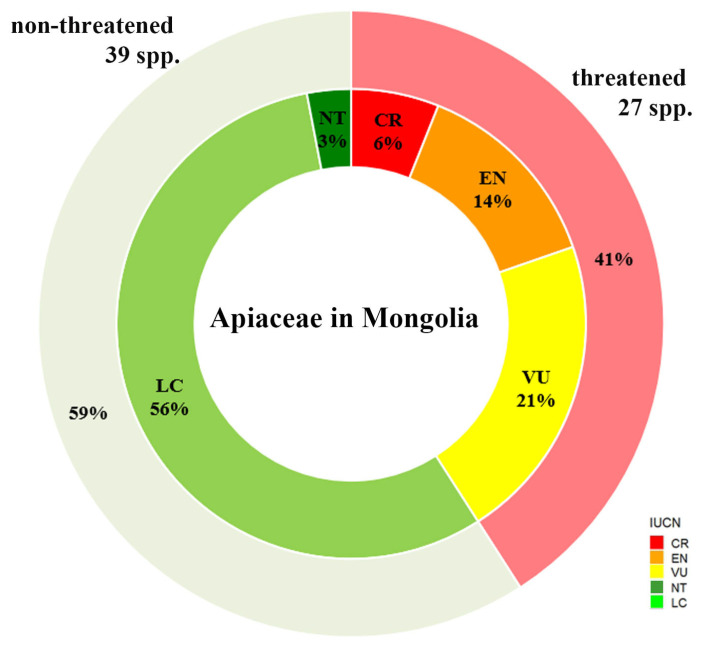
The national Red List statuses of Apiaceae species in Mongolia according to IUCN Criterion B.

The national IUCN Red List statuses of the 66 taxa were evaluated under Criterion B at the national level (Figure 1, Table A1). Among these, 27 taxa were classified as threatened, including 4 Critically Endangered (CR), 9 Endangered (EN), and 14 Vulnerable (VU) species. The remaining 39 species were assessed as non-threatened, comprising 2 Near-Threatened (NT) species and 37 species of Least Concern (LC). 

We obtained high-quality photographs of these species from our own field surveys and the iNaturalist platform (Appendix B, Figure A1, Figure A2 and Figure A3). Approximately 90% of all species were adequately photographed in Mongolia. In addition, we generated distribution maps for 66 taxa based on all occurrences, as shown in the Appendix C (Figure A4, Figure A5, Figure A6, Figure A7, Figure A8, Figure A9, Figure A10, Figure A11, Figure A12, Figure A13, Figure A14, Figure A15, Figure A16, Figure A17, Figure A18, Figure A19, Figure A20, Figure A21, Figure A22, Figure A23, Figure A24, Figure A25, Figure A26, Figure A27, Figure A28, Figure A29, Figure A30, Figure A31, Figure A32, Figure A33, Figure A34, Figure A35, Figure A36, Figure A37, Figure A38, Figure A39, Figure A40, Figure A41, Figure A42, Figure A43, Figure A44, Figure A45, Figure A46, Figure A47, Figure A48, Figure A49, Figure A50, Figure A51, Figure A52, Figure A53, Figure A54, Figure A55, Figure A56, Figure A57, Figure A58, Figure A59, Figure A60, Figure A61, Figure A62, Figure A63, Figure A64, Figure A65, Figure A66, Figure A67, Figure A68, Figure A69 and Figure A70). Based on the distribution map results, the majority of the species were collected prior to 2000. Finally, we identified several new locations of Apiaceae species for the phytogeographical regions which are marked as plus (Appendix A). 

### 2.2. Distribution Patterns of Species Richness 

**Figure 2:**
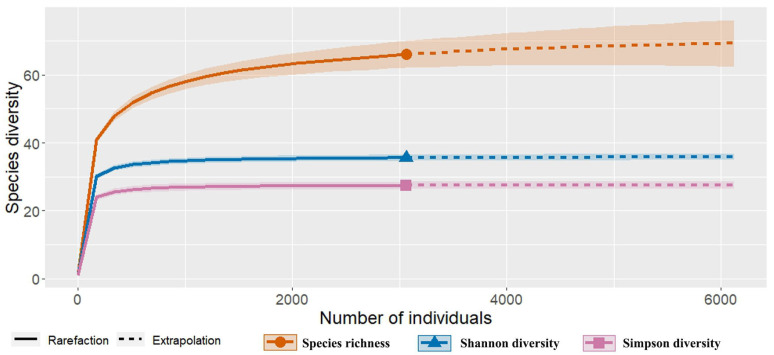
The species occurrences of the family Apiaceae in Mongolia. The shading surrounding the line corresponds to 95% confidence intervals.

We assessed the species diversity and Shannon and Simpson diversity indices of Apiaceae based on all occurrence records. In general, the Hill diversity estimates indicated that the sampling conducted in Mongolia adequately covered species occurrence (Figure 2). According to the rarefaction curve, the numbers of both the species and individuals were sufficient, as indicated by the saturated lines of the Shannon (blue) and Simpson (pink) diversity indices (Figure 2). However, the species richness (orange line) exhibited a slight tendency to increase with the number of individuals.

**Figure 3:**
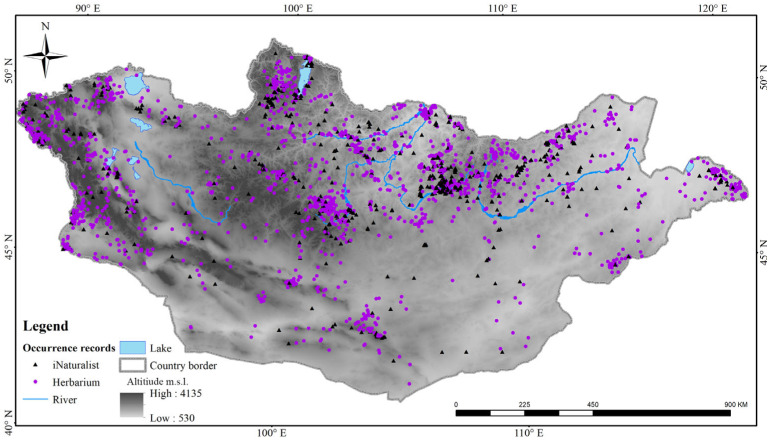
The species occurrences of Apiaceae in Mongolia.

A total of 3062 unique records were obtained, including 2015 and 1047 records from herbaria and the iNaturalist platform, respectively (Figure 3). The majority of occurrences were in mountainous areas of Mongolia such as the Altai, Khangai, Khentii and Khuvsgul mountains. Most records were from mountain steppe, forest steppe, and steppe habitats.

**Figure 4:**
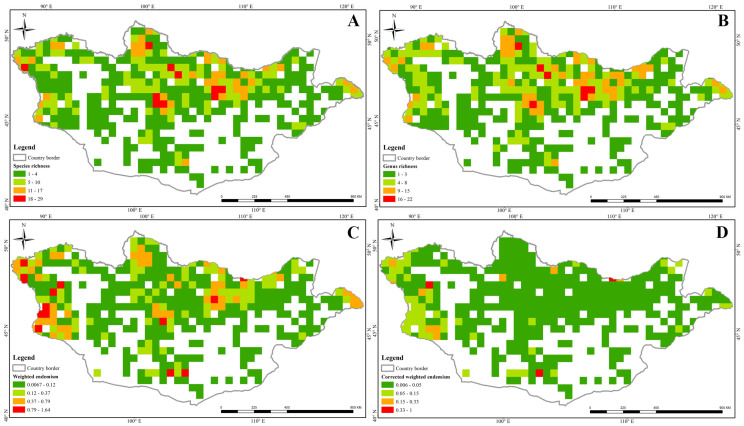
Indicators of the species diversity of Apiaceae in Mongolia. (**A**) Species richness; (**B**) genus richness; (**C**) weighted endemism; (**D**) corrected weighted endemism.

Of the 715 grid cells covering Mongolia, 408 contained at least one species record. We calculated the SR, WE, and CWE for all occurrences across Mongolia. Our data collection was highly comprehensive because of our exhaustive search of all available data sources. According to the SR parameter, all species were unevenly distributed throughout the country, except in the southern and western regions (Figure 4A). We identified 10 grid cells that exhibited a high level of species richness, each containing 18–29 Apiaceae species. In addition, we identified 36 grid cells with 11–17 species (Figure 4A). We also considered genus richness: seven grids had high genus richness, with 16–22 genera in each grid (Figure 4B). Twelve grid cells had high WE; these were distributed in the Mongolian Altai, Khentii, Khangai, and Gurvan Saikhan mountain ranges (Figure 4C). Only three cells featuinf a high CWE index were identified, and these were scattered throughout the Khentii, Jargalant Khairkhan, and Gurvan Saikhan mountain ranges (Figure 4D).

**Figure 5:**
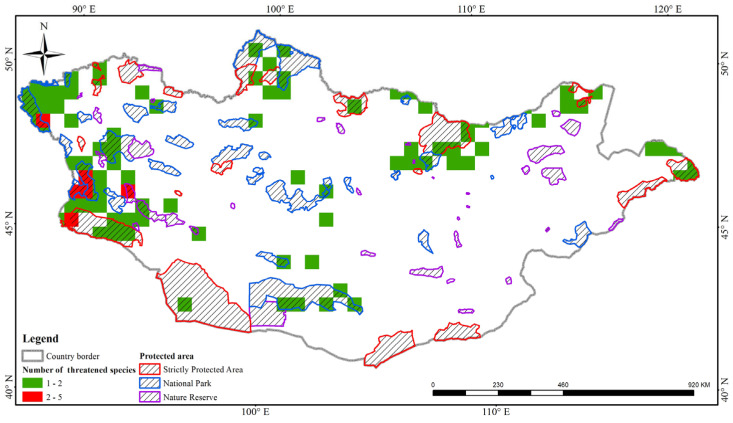
Overlap between threatened species richness (comprising CR, EN, and VU species) and protected areas in Mongolia.

In addition, we examined the species diversity of threatened Apiaceae species overlapping with all protected areas in Mongolia. A total of 92 grid cells contained at least one threatened species. Among these, six grid cells had two to five threatened Apiaceae species that occurred in the Altai-Dzungarian mountain range of Mongolia (Figure 5). The majority of the threatened species richness was covered by formally protected areas or national parks (Figure 5). For example, the grid cells containing two to five threatened Apiaceae species were distributed within the Great Gobi B, Altai Tavan Bogd, and Bulgan-Gol Ikh Ongog national parks in western Mongolia (Figure 5).

**Figure 6:**
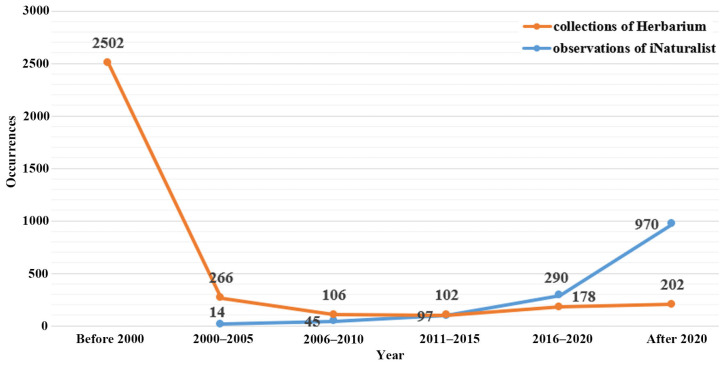
The number of occurrences of Apiaceae species in Mongolia collected from herbariums and the iNaturalist.

We compared the herbarium data and iNaturalist observations of Apiaceae, focusing on the collection period (Figure 6). The majority of herbarium specimens were collected prior to 2000, with relatively few collections being made since. However, the number of iNaturalist observations is increasing each year because of the number of citizen scientists uploading photo observations to the Flora of Mongolia project (https://www.inaturalist.org/projects/flora-of-mongolia, accessed on 12 March 2024).

## 3. Discussion

The Red List serves as a valuable tool for conservation planning and decision making. It helps governments, NGOs, and other stakeholders identify areas and species in need of protection, as well as to develop conservation strategies and policies [27]. In addition, the Red List is a crucial tool for assessing the status of species worldwide, guiding conservation action by informing prioritization processes and promoting the conservation of biodiversity for future generations. Therefore, several previous publications have assessed the conservation status of vascular plants at the global level using Criterion B [4,9,28,29]. In this study, we evaluated the national conservation status of all Apiaceae species using the ConR package in accordance with IUCN Criterion B. An advantage of Criterion B is that can be used to assess the status of species with unknown population sizes or rates of decline.

In recent decades, a number of researchers have been conducting species pattern analyses on various plants groups in the world. This is because species richness analyses help to inform the future conservation and management of protected areas and land, respectively [3,4]. However, only a few studies have applied species richness analyses and conservation status assessments to vascular plants and fungi in Mongolia [30,31]. For example, Baasanmunkh et al. [30] studied the diversity of Orchidaceae, for which a number of high diversity grids are not covered by protected areas. In this study, we analyzed the species patterns of Apiaceae using three different indices. In general, our data collection was the most comprehensive because of our exhaustive search of all available data sources. Based on species pattern analysis, we identified four highly diverse hotspots in the central, western, and northern parts of Mongolia (Figure 4A). In addition, we performed species richness analyses solely for threatened species, such as CR, EN, and VU species, in accordance with our own recently developed assessments (Figure 4). Fortunately, the majority of the threatened species richness was covered by formally protected areas or national parks (Figure 4). For example, two to five threatened Apiaceae species were distributed within the Great Gobi B, Altai Tavan Bogd, and Bulgan-Gol Ikh Ongog national parks in western Mongolia (Figure 4). Our results on species richness and conservation status, along with those on Orchidaceae [30] and fungi [31], are important fundamental pieces of research for future conservation and land management efforts in Mongolia.

Interestingly, we found additional occurrence records for several threatened species that had previously been noted in only one or a few locations in Mongolia. For example, the nationally Endangered *Sajanella monstrosa* (Willd.) Soják was previously known only in the Khentei region of northern Mongolia; however, we discovered it in the Khuvsgul region in north-western Mongolia in 2020 [27]. Furthermore, several important medicinal Apiaceae species have been identified in Mongolia. For instance, *Saposhnikovia divaricata* (Turcz.) Schischk. (Apiaceae) is a widely recognized traditional medicinal plant [32], but its distribution is decreasing because of illegal collection. It should be noted that most records are from prior to 2000, with there being a minimal number of recent collections. This could be attributed to the potential extinction of numerous individuals in the wild due to climate change or overgrazing across Mongolia. If it is indeed the case that some occurrence records are now obsolete due to species becoming locally extirpated, we may have overestimated EOO and/or AOO for some species, resulting in species being assigned a lower threat status than they deserve in reality. Thus, the status of the Apiaceae species in Mongolia could be worse than is currently believed. Hopefully, this will become clearer as the number of citizen scientists increases.

## 4. Materials and Methods

### 4.1. Data Collection

Occurrence data for each species were gathered from several sources: (1) our field data collected over 15 years; (2) available herbarium data from ALTB, E, GAT, GFW, HAL, INM, K, LE, MW, MEL, NS, NSK, OSBU, PL, UBA, UBU, and US [33]; (3) the Flora of Mongolia project on the iNaturalist platform (https://www.inaturalist.org/projects/flora-of-mongolia, accessed on 12 March 2024); (4) the Global Biodiversity Information Facility (GBIF) platform and the Virtual Guide to Mongolia [34,35]. We collected data on approximately 4752 occurrences of Apiaceae in Mongolia. Among these, over 2000 occurrences are not available online, being stored in the UBA and UBU herbariums in Mongolia. We recorded 4752 occurrences of Apiaceae taxa based on all available data. We subsequently removed 1690 duplicate and non-georeferenced records. In total, 3062 occurrences were analyzed.

**Figure A4:**
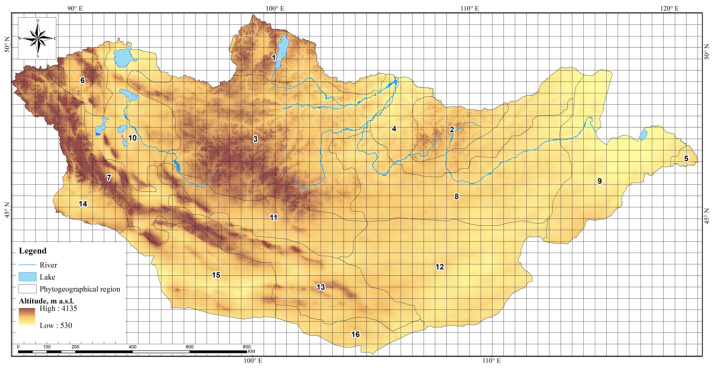
Map of general phytogeographical regions of Mongolia (by Grubov 1982). (1—Khuvsgul, 2—Khentei, 3—Khangai, 4—Mongolian Dauria, 5—Foothills of Great Khyangan, 6—Khovd, 7—Mongolian Altai, 8—Middle Khalkh, 9—East Mongolia, 10—Depression of Great Lakes, 11—Valley of Lakes, 12—East Gobi, 13—Gobi Altai, 14—Dzungarian Gobi, 15—Transaltai, 16—Alashan Gobi).

For the species distribution mapping, we used topographic maps depicting phytogeographical regions and an altitude range of approximately 520–4300 m a.s.l. (Figure A4) based on [25]. This distribution map was derived from [11,25], with modifications. Distribution points are represented by three different symbols, which are summarized in Table 1.

### 4.2. Conservation Status Assessment

Taxa were assessed under Red List Criterion B in accordance with the IUCN national Red List guidelines [36]. Criterion B uses geographic range (EOO or AOO) and evidence of population decline, fragmentation, or fluctuations to assess extinction risk. The EOO and AOO were estimated using the ConR package [37] in R v.4.0.4 programming software [38]. The AOO was estimated based on a user-defined grid cell of 2 km^2^, as recommended by the IUCN [29]. We did not assess the three non-native Apiaceae species found in Mongolia.

### 4.3. Distribution Patterns of Species Richness

First, we analyzed Hill numbers (including q = 0, 1, and 2) using the iNEXT package [39]. For instance, the Hill number q = 0 corresponds to species richness, q = 1 reflects the effective number of species (which is the exponent of Shannon diversity), and q = 2 denotes the number of dominant species (inverse Simpson diversity; [40]). Second, we created a grid net for Mongolia with a spatial resolution of 0.5° × 0.5° grid size (equivalent to approximately 50 × 50 squares) using the FishNet tool in ArcGIS 10.3 [41]. Mongolia was partitioned into 715 grid cells. We estimated three diversity indices (species richness (SR), weighted endemism (WE), and corrected weighted endemism (CWE)) using Biodiverse v.4.1 software [42]. WE is estimated by considering the presence or absence of a species within a cell, whereas CWE is determined by calculating the proportion of endemic species within a cell in relation to the total endemic species richness of the cell [42]. Furthermore, we used geographic information system data regarding protected areas obtained from the World Database on Protected Areas (WDPA; https://protectedplanet.net/, accessed on 12 March 2024) to determine the extent to which threatened species were covered by protected areas. We excluded natural monuments, a category of protected areas in Mongolia, because they are typically geographically restricted to small areas and primarily focus on preserving historical and cultural heritage.

## 5. Conclusions

In this study, we conducted a comprehensive evaluation of the national Red List statuses of 66 taxa in Mongolia according to IUCN Criterion B, shedding light on the conservation statuses of these species. Our findings revealed that a significant portion of these taxa, totaling 27 and 39 species, are classified as threatened and non-threatened, respectively. Our efforts resulted in comprehensive distribution maps for all 66 taxa. Moreover, our study assessed species diversity and distribution across Mongolia, utilizing various diversity indices and occurrence records. Notably, we identified several high biodiversity hotspots, particularly in mountainous areas such as the Altai, Khangai, Khentii, and Khuvsgul mountains. Finally, we compared herbarium data and iNaturalist observations of Apiaceae, revealing interesting trends in collection periods and observation frequencies. While herbarium specimens were predominantly collected before 2000, the number of iNaturalist observations has been steadily increasing since then, indicating the growing contribution of citizen scientists to biodiversity research. Overall, our study provides valuable insights into the conservation status, distribution patterns, and biodiversity of Apiaceae species in Mongolia, laying a foundation for targeted conservation efforts and further research endeavors in the region.

## Figures and Tables

**Table 1:** Mapping symbols used in the distribution maps.

Symbols	Description	Brief Explanation
●	Before 2000	Based on herbarium collections
○	After 2000	
▲	All other	Based on other sources: literature and observations from iNaturalist (since January 2019)

**Figure A1:**
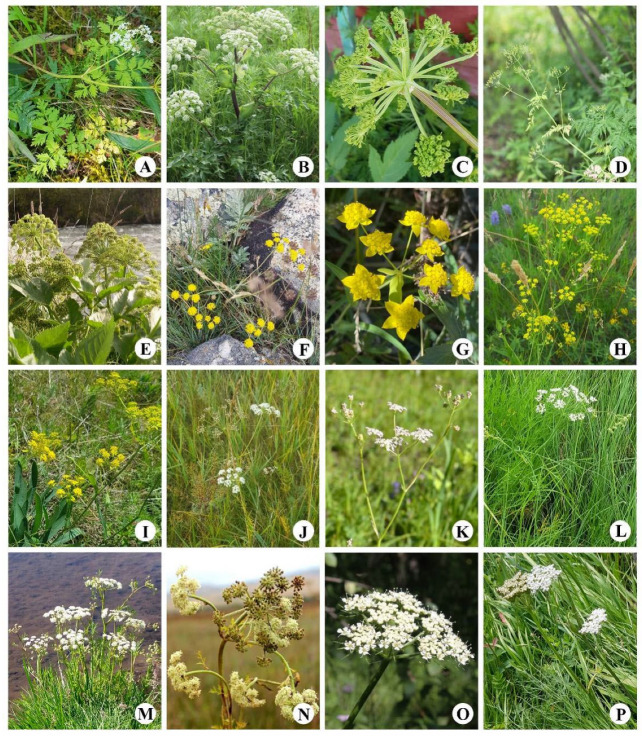
Some species of Apiaceae in Mongolia. (**A**) *Aegopodium alpestre*. (**B**) *Angelica dahurica*. (**C**) *Angelica sylvestris*. (**D**) *Anthriscus sylvestris*. (**E**) *Archangelica decurrens*. (**F**) *Bupleurum bicaule*. (**G**) *Bupleurum multinerve*. (**H**) *Bupleurum scorzonerifolium*. (**I**) *Bupleurum sibiricum*. (**J**) *Carum buriaticum*. (**K**) *Carum carvi*. (**L**) *Cenolophium denudatum*. (**M**) *Cicuta virosa*. (**N**) *Cnidium dahuricum*. (**O**) *Cnidium monnieri*. (**P**) *Conioselinum longifolium*. Photos: (**A**,**C**,**E**–**N**,**P** by M.Urgamal), (**B**,**D** by B.Oyuntsetseg) and (**O** by Ch.Javzandolgor).

**Figure A2:**
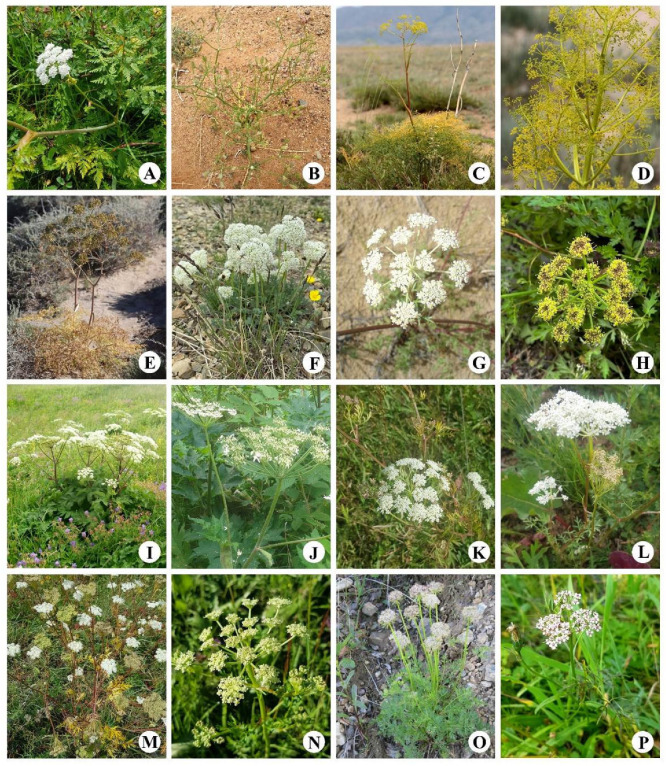
Some species of Apiaceae in Mongolia. (**A**) *Conioselinum tataricum*. (**B**) *Ferula bungeana*. (**C**) *Ferula dshaudshamyr*. (**D**) *Ferula ferulioides*. (**E**) Ferula songarica. (**F**) Ferulopsis hystrix. (**G**) *Haloselinum falcaria*. (**H**) *Hansenia mongholica*. (**I**) *Heracleum dissectum*. (**J**) *Heracleum sibiricum*. (**K**) *Kadenia salina*. (**L**) *Kitagawia baicalensis*. (**M**) *Kitagawia terebinthacea*. (**N**) *Lithosciadium multicaule*. (**O**) *Neogaya simplex*. (**P**) *Ostericum tenuifolium*. Photos: (**A**,**B**,**G**,**I**–**P** by M.Urgamal), and (**C**–**F**,**H** by B.Oyuntsetseg).

**Figure A3:**
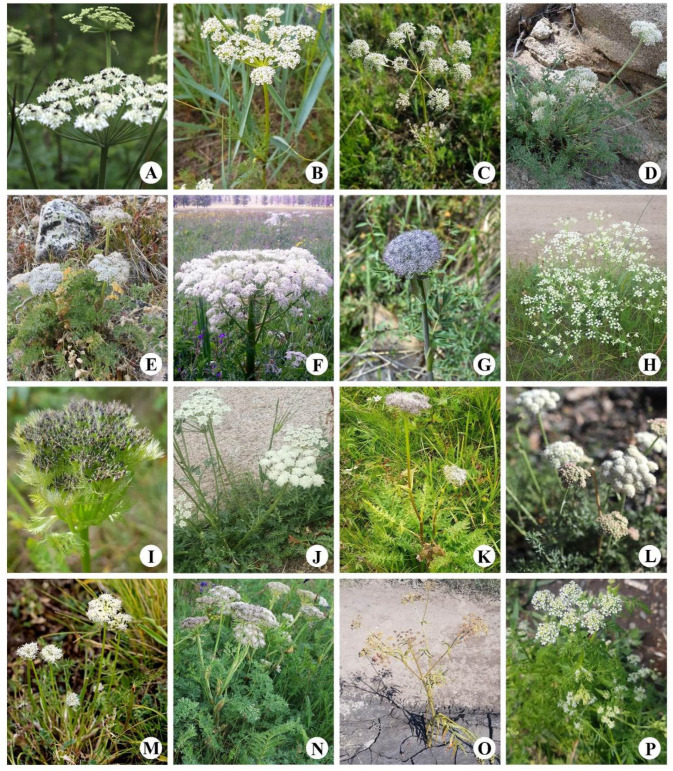
Some species of Apiaceae in Mongolia. (**A**) *Paraligusticum discolor*. (**B**) *Peucedanum puberulum*. (**C**) *Peucedanum vaginatum*. (**D**) *Phlojodicarpus sibiricus*. (**E**) *Phlojodicarpus villosus*. (**F**) *Pleurospermum uralense*. (**G**) *Sajanella monstrosa*. (**H**) *Saposhnikovia divaricata*. (**I**) *Schulzia crinita*. (**J**) *Seseli buchtormense*. (**K**) *Seseli condensatum*. (**L**) *Seseli eriocarpum*. (**M**) *Seseli glabratum*. (**N**) *Seseli seseloides*. (**O**) *Sium suave*. (**P**) *Sphallerocarpus gracilis*. Photos: (**A**,**L**,**M** by B.Oyuntsetseg), (**B**–**D**,**F**,**H**–**K**,**N**–**P** by M.Urgamal), (**E** by Ch.Javzandolgor) and (**G** by Sh.Baasanmunkh).

**Figure A5:**
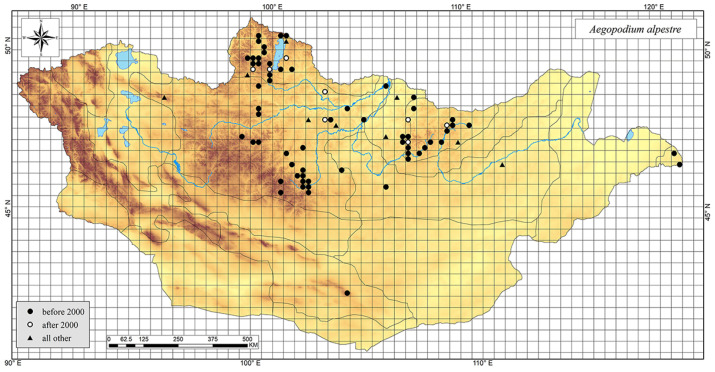
Distribution of *Aegopodium alpestre* in Mongolia.

**Figure A6:**
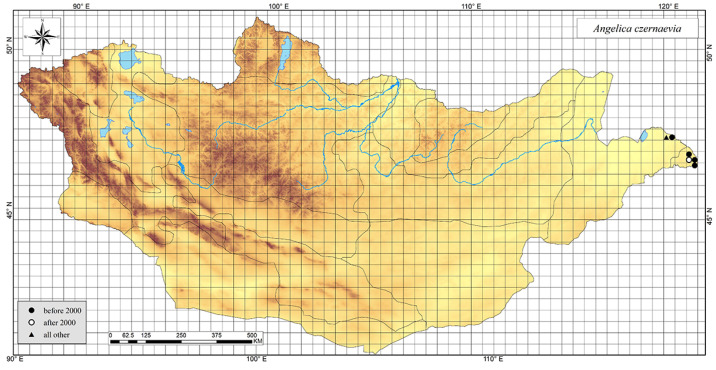
Distribution of *Angelica czernaevia* in Mongolia.

**Figure A7:**
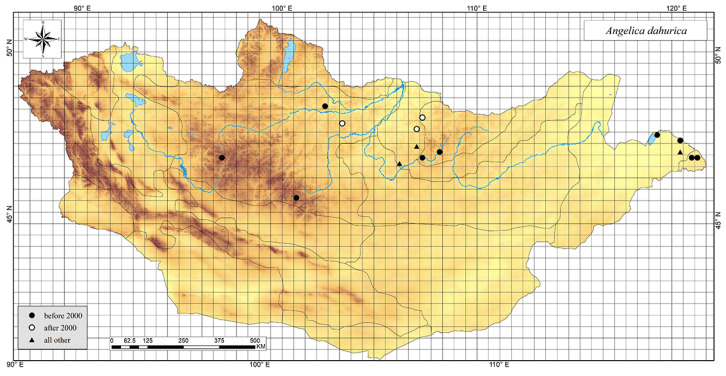
Distribution of *Angelica dahurica* in Mongolia.

**Figure A8:**
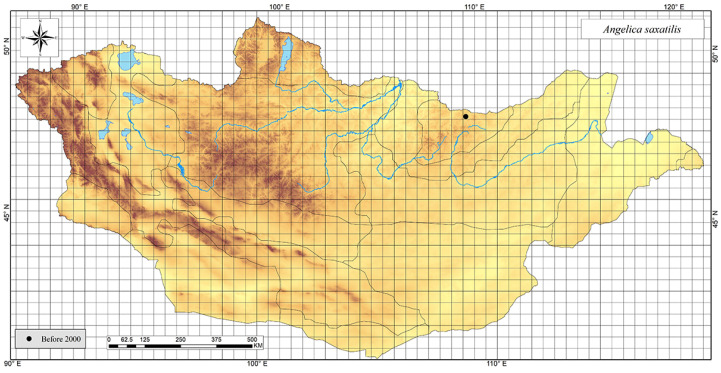
Distribution of *Angelica saxatilis* in Mongolia.

**Figure A9:**
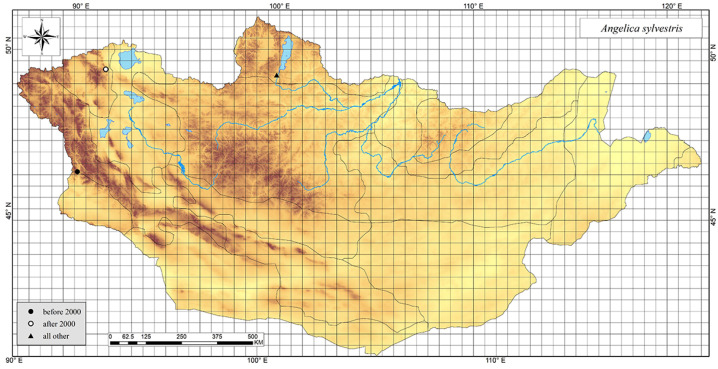
Distribution of *Angelica sylvestris* in Mongolia.

**Figure A10:**
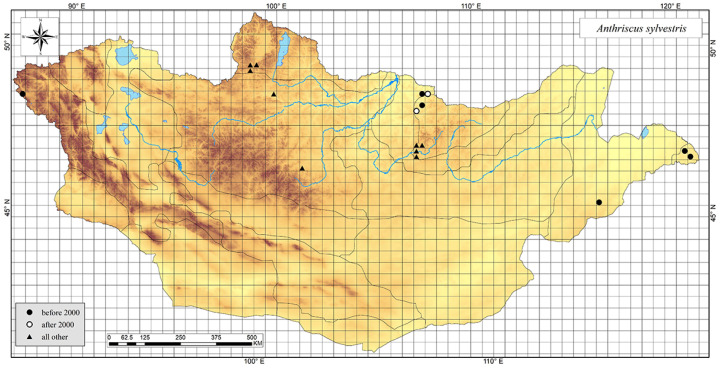
Distribution of *Anthriscus sylvestris* in Mongolia.

**Figure A11:**
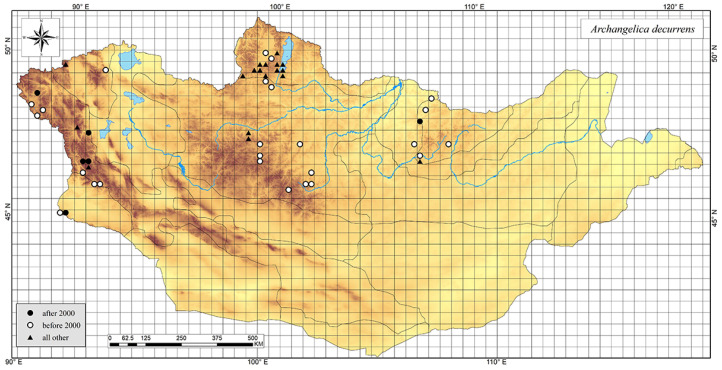
Distribution of *Archangelica decurrens* in Mongolia.

**Figure A12:**
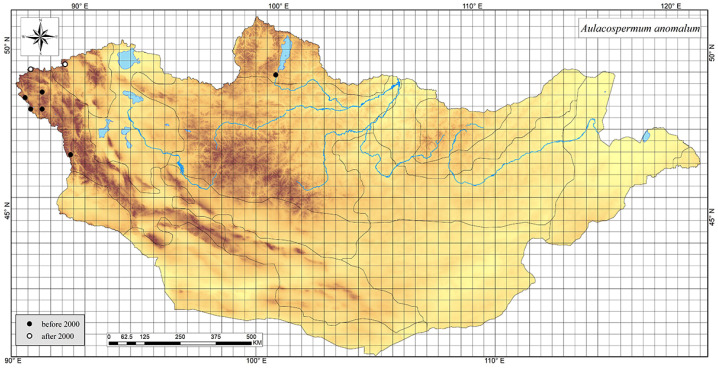
Distribution of *Aulacospermum anomalum* in Mongolia.

**Figure A13:**
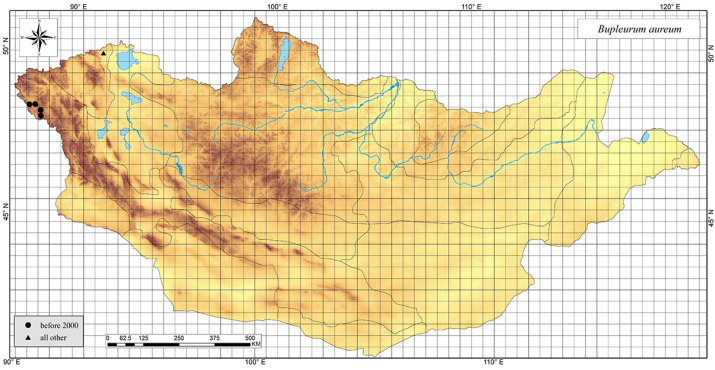
Distribution of *Bupleurum aureum* in Mongolia.

**Figure A14:**
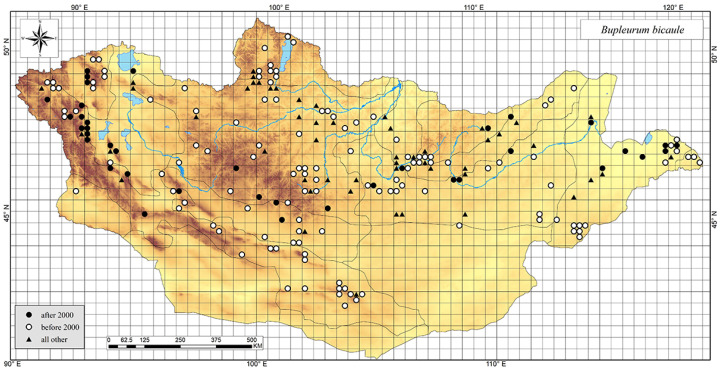
Distribution of *Bupleurum bicaule* in Mongolia.

**Figure A15:**
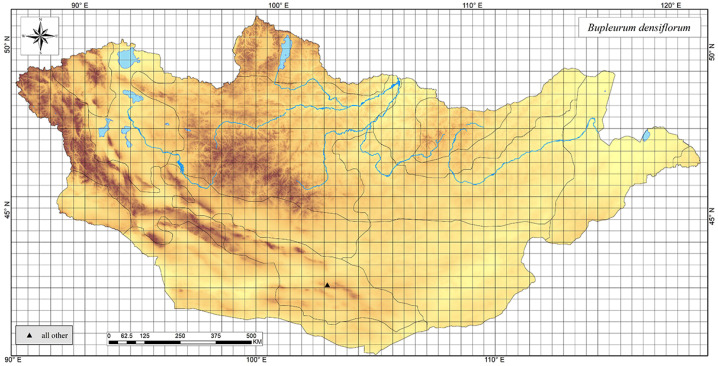
Distribution of *Bupleurum densiflorum* in Mongolia.

**Figure A16:**
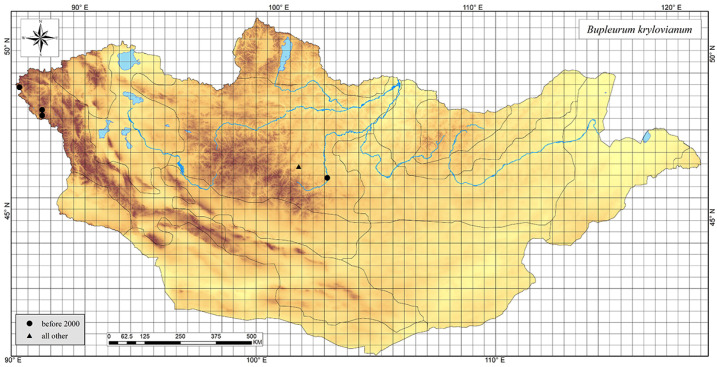
Distribution of *Bupleurum krylovianum* in Mongolia.

**Figure A17:**
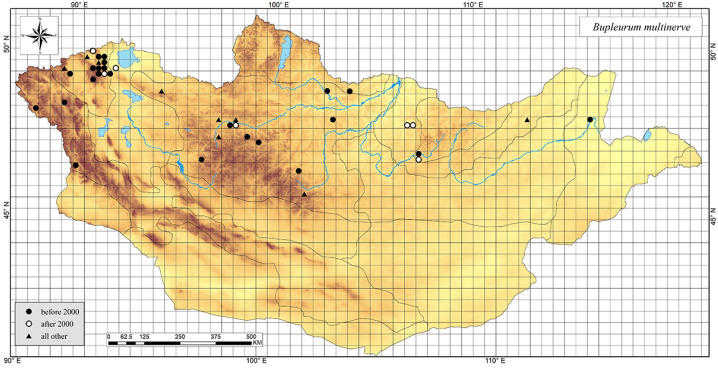
Distribution of *Bupleurum multinerve* in Mongolia.

**Figure A18:**
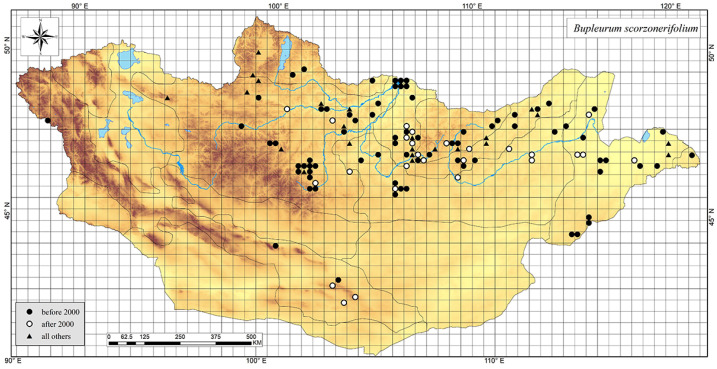
Distribution of *Bupleurum scorzonerifolium* in Mongolia.

**Figure A19:**
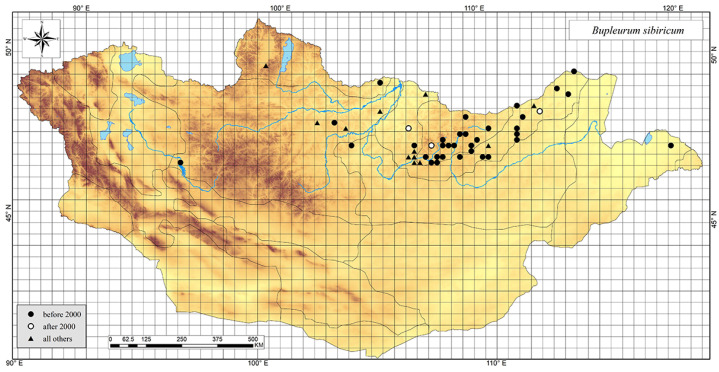
Distribution of *Bupleurum sibiricum* in Mongolia.

**Figure A20:**
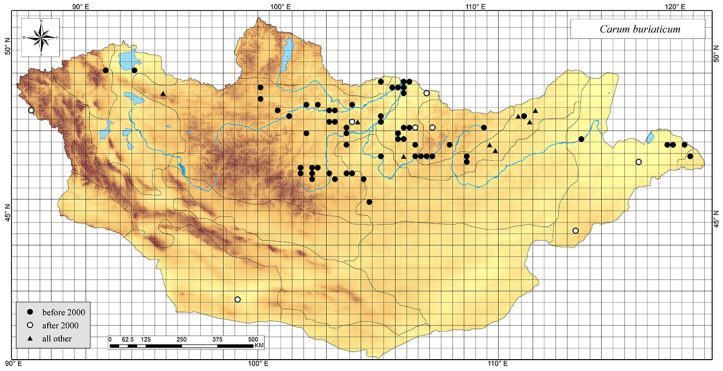
Distribution of *Carum buriaticum* in Mongolia.

**Figure A21:**
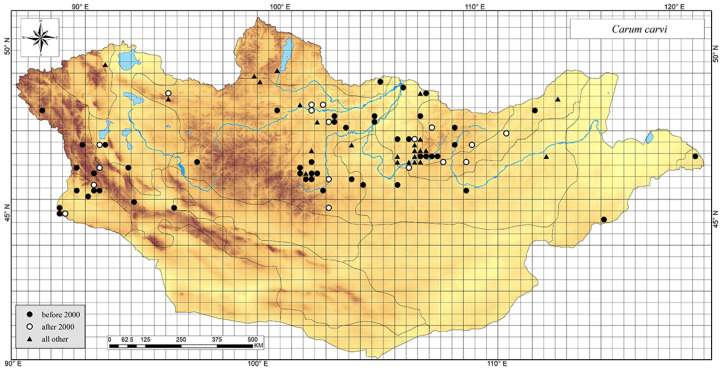
Distribution of *Carum carvi* in Mongolia.

**Figure A22:**
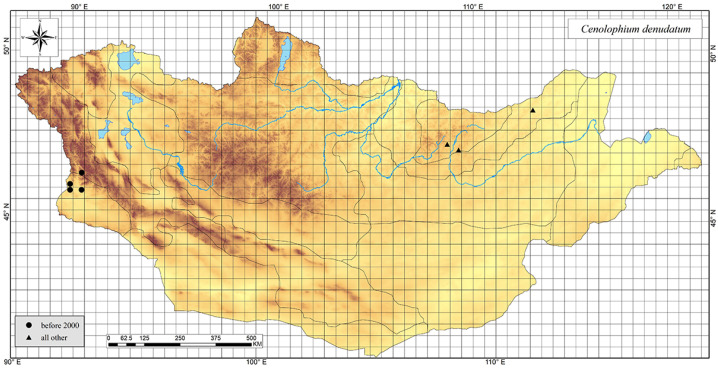
Distribution of *Cenolophium denudatum* in Mongolia.

**Figure A23:**
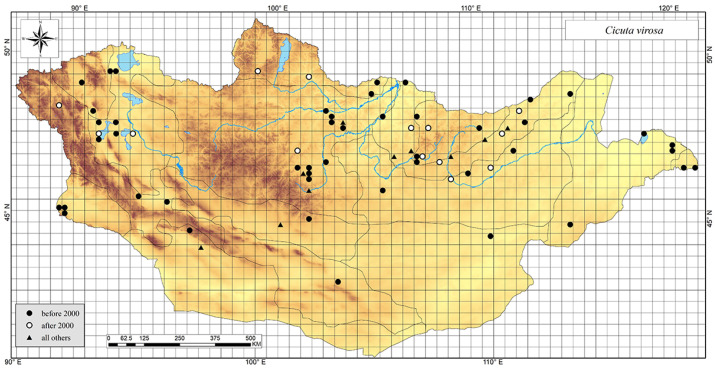
Distribution of *Cicuta virosa* in Mongolia.

**Figure A24:**
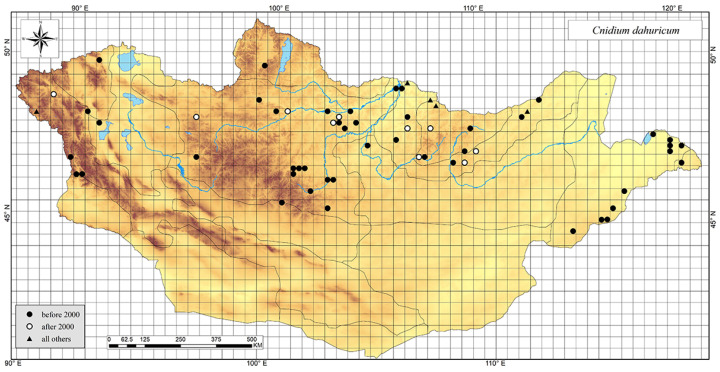
Distribution of *Cnidium dahuricum* in Mongolia.

**Figure A25:**
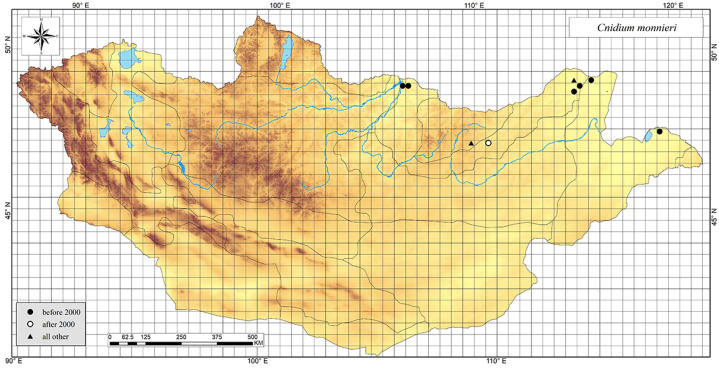
Distribution of *Cnidium monnieri* in Mongolia.

**Figure A26:**
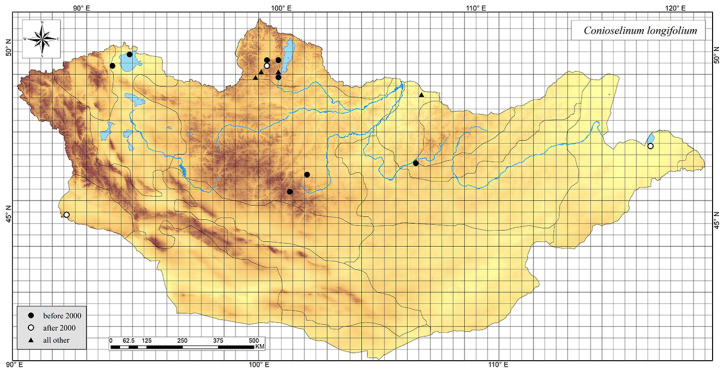
Distribution of *Conioselinum longifolium* in Mongolia.

**Figure A27:**
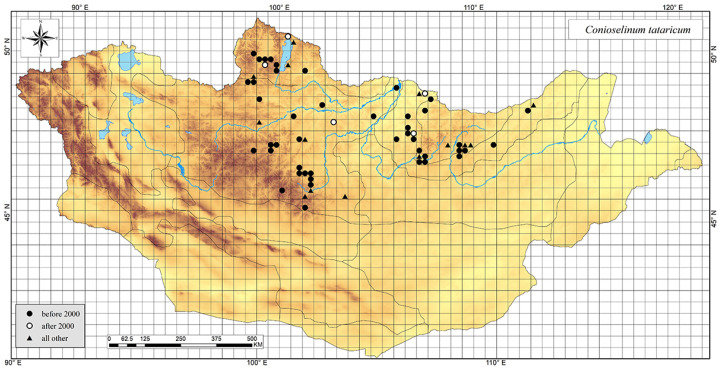
Distribution of *Conioselinum tataricum* in Mongolia.

**Figure A28:**
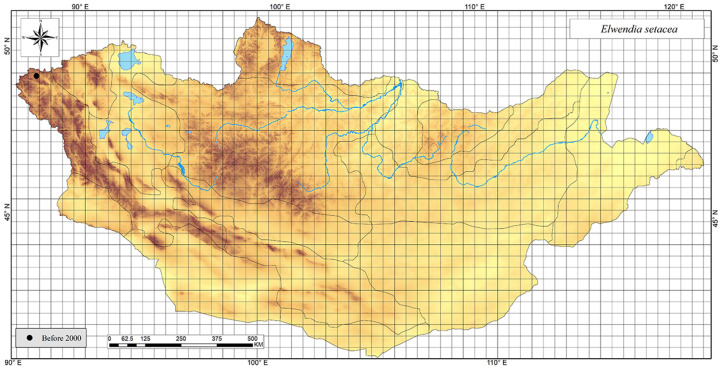
Distribution of *Elwendia setacea* in Mongolia.

**Figure A29:**
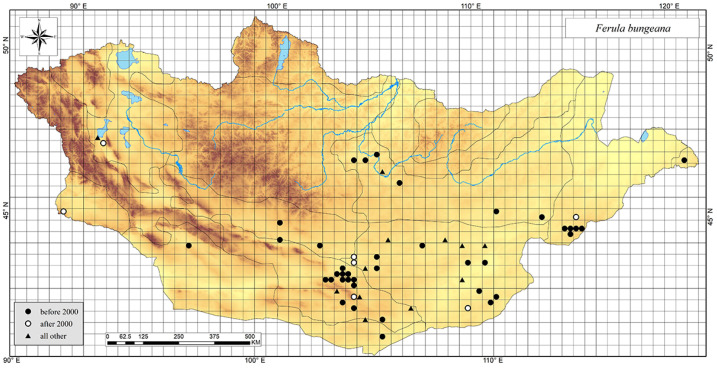
Distribution of *Ferula bungeana* in Mongolia.

**Figure A30:**
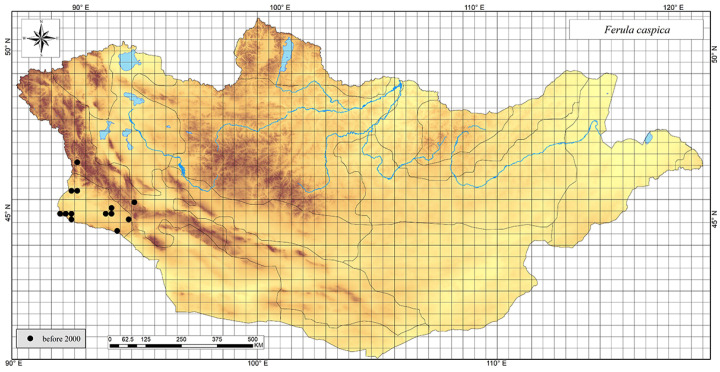
Distribution of *Ferula caspica* in Mongolia.

**Figure A31:**
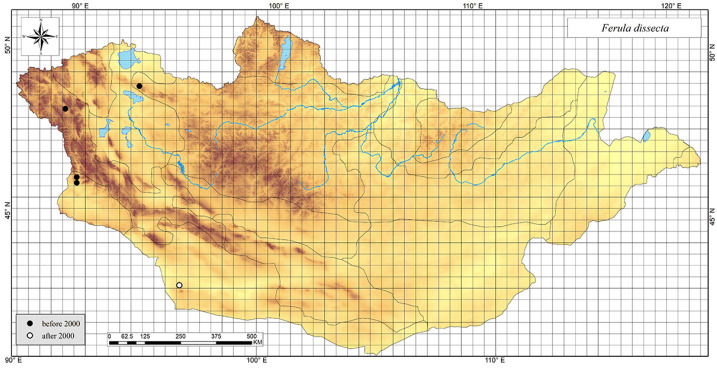
Distribution of *Ferula dissecta* in Mongolia.

**Figure A32:**
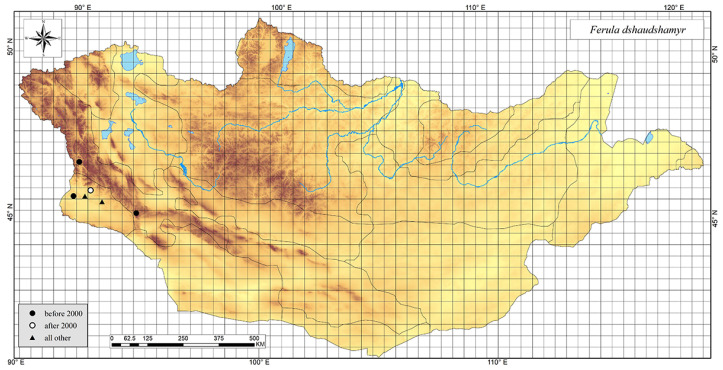
Distribution of *Ferula dshaudshamyr* in Mongolia.

**Figure A33:**
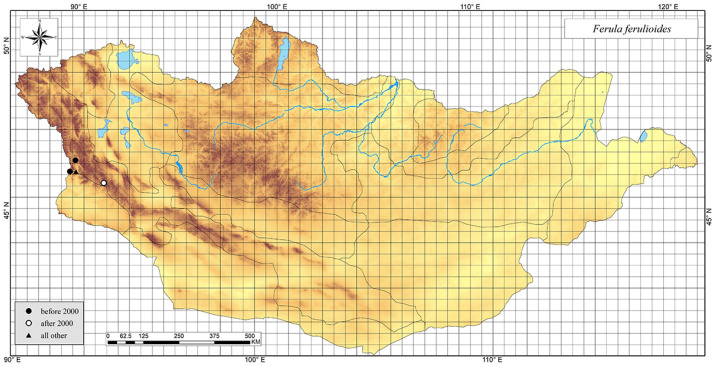
Distribution of *Ferula ferulioides* in Mongolia.

**Figure A34:**
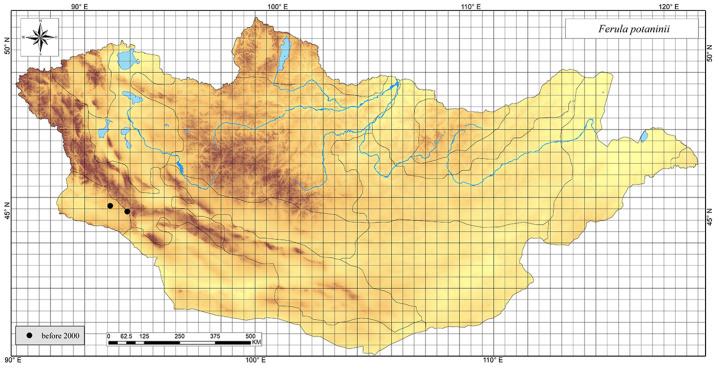
Distribution of *Ferula potaninii* in Mongolia.

**Figure A35:**
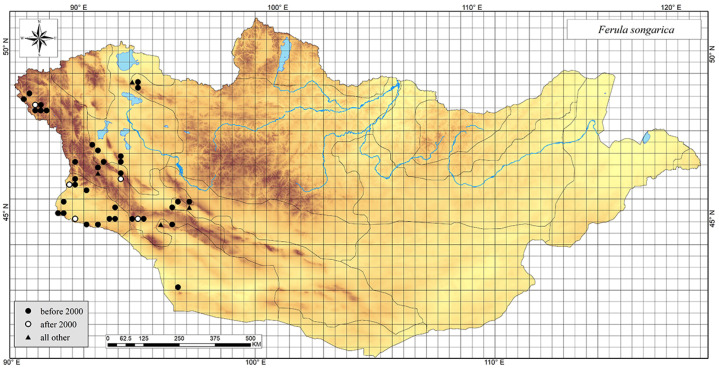
Distribution of *Ferula songarica* in Mongolia.

**Figure A36:**
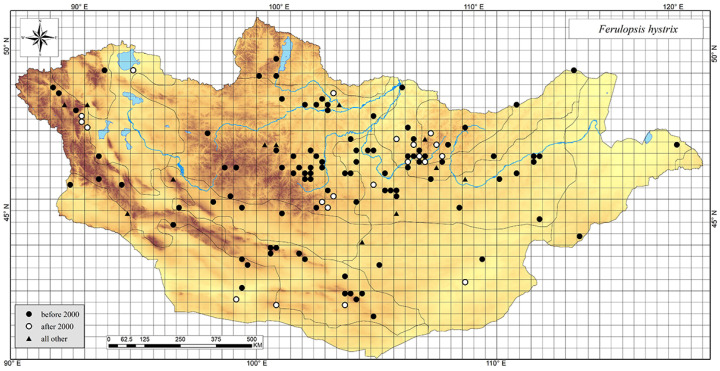
Distribution of *Ferulopsis hystrix* in Mongolia.

**Figure A37:**
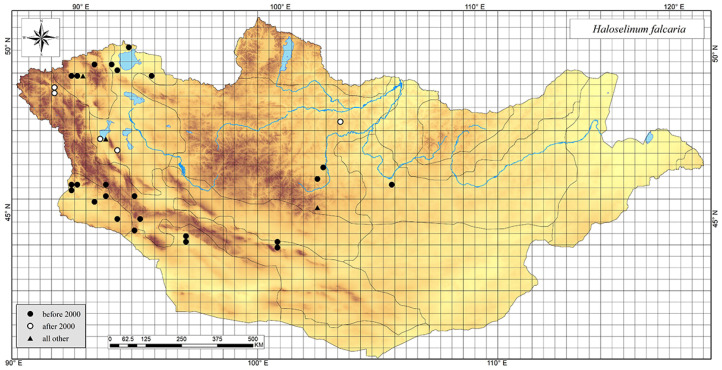
Distribution of *Haloselinum falcaria* in Mongolia.

**Figure A38:**
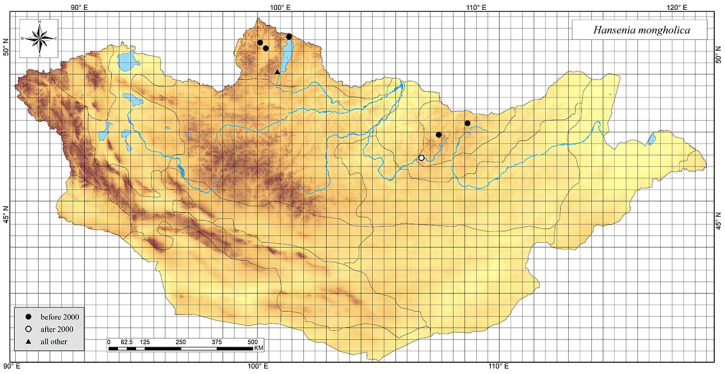
Distribution of *Hansenia mongholica* in Mongolia.

**Figure A39:**
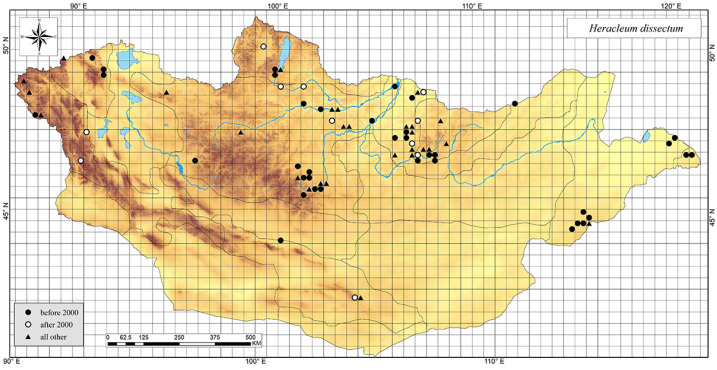
Distribution of *Heracleum dissectum* in Mongolia.

**Figure A40:**
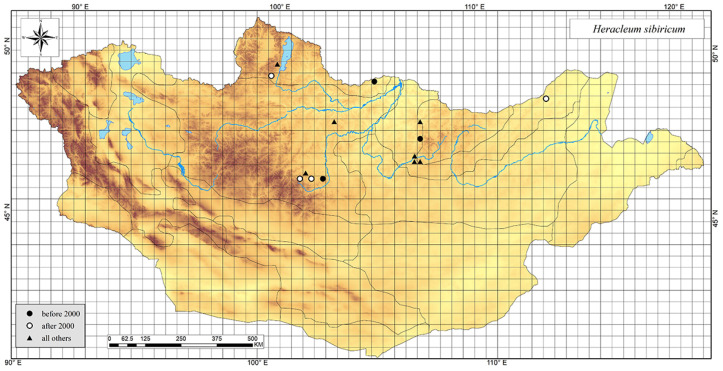
Distribution of *Heracleum sibiricum* in Mongolia.

**Figure A41:**
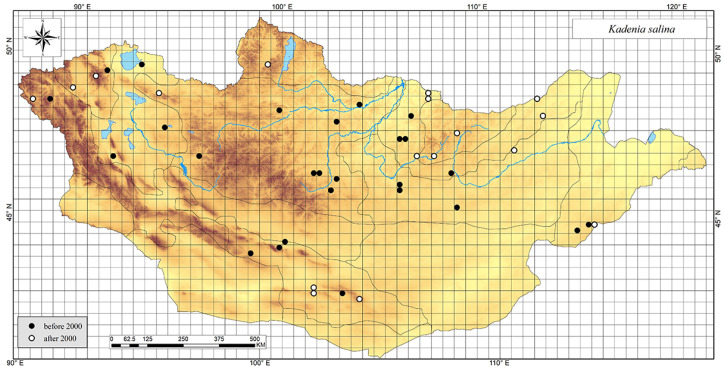
Distribution of *Kadenia salina* in Mongolia.

**Figure A42:**
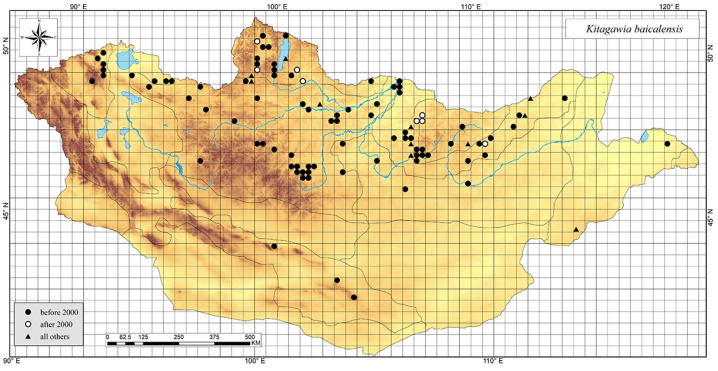
Distribution of *Kitagawia baicalensis* in Mongolia.

**Figure A43:**
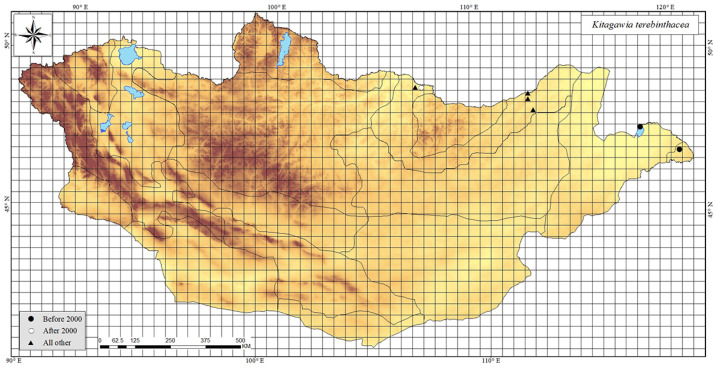
Distribution of *Kitagawia terebinthacea* in Mongolia.

**Figure A44:**
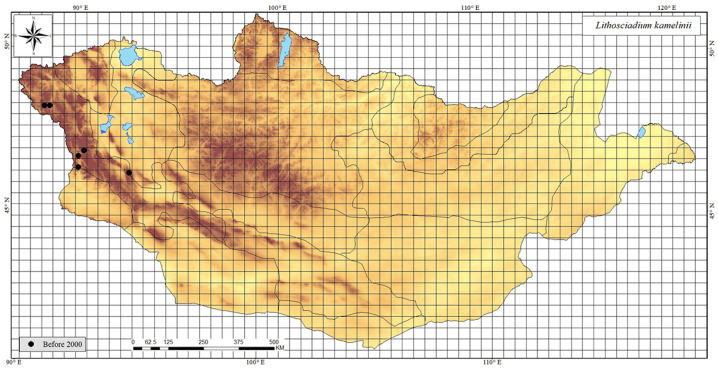
Distribution of *Lithosciadium kamelinii* in Mongolia.

**Figure A45:**
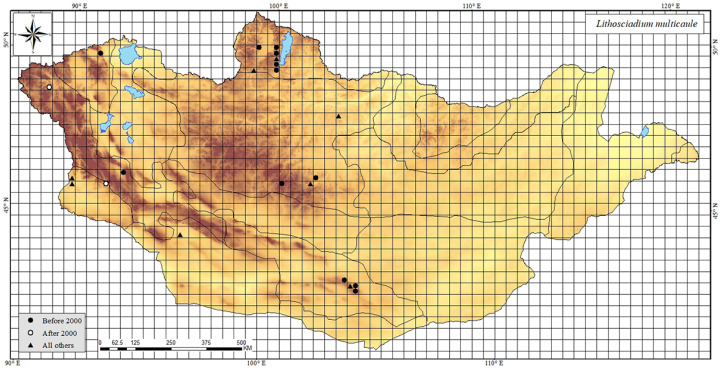
Distribution of *Lithosciadium multicaule* in Mongolia.

**Figure A46:**
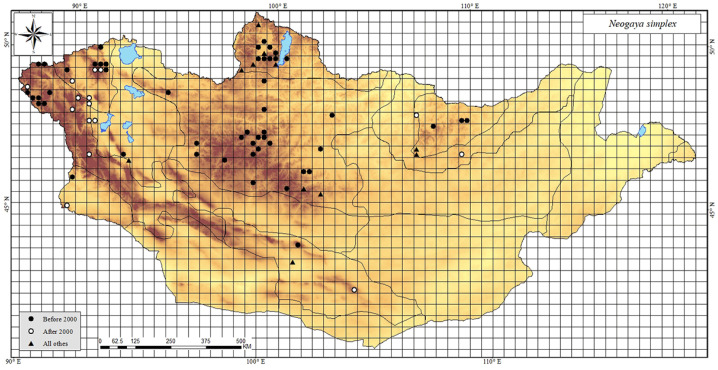
Distribution of *Neogaya simplex* in Mongolia.

**Figure A47:**
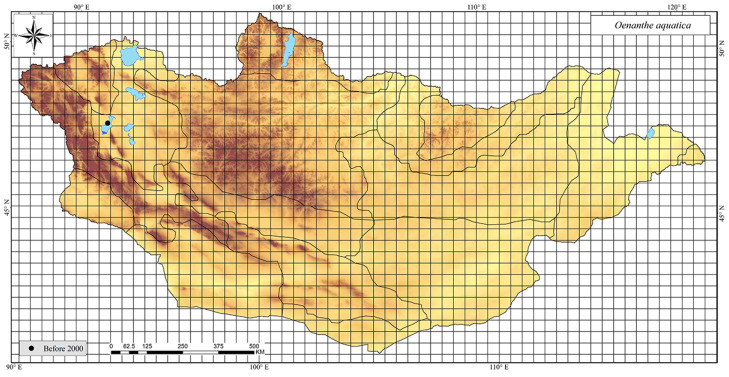
Distribution of *Oenanthe aquatica* in Mongolia.

**Figure A48:**
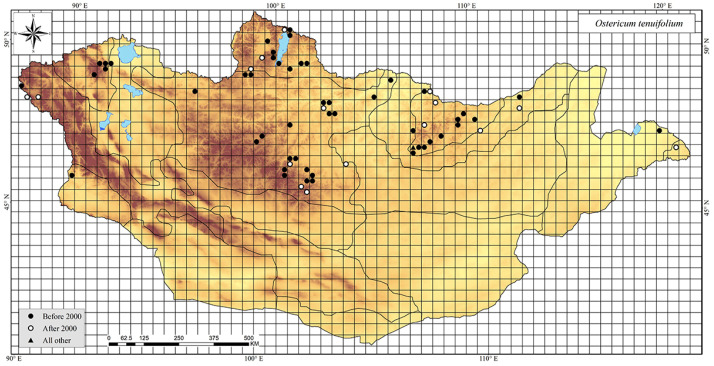
Distribution of *Ostericum tenuifolium* in Mongolia.

**Figure A49:**
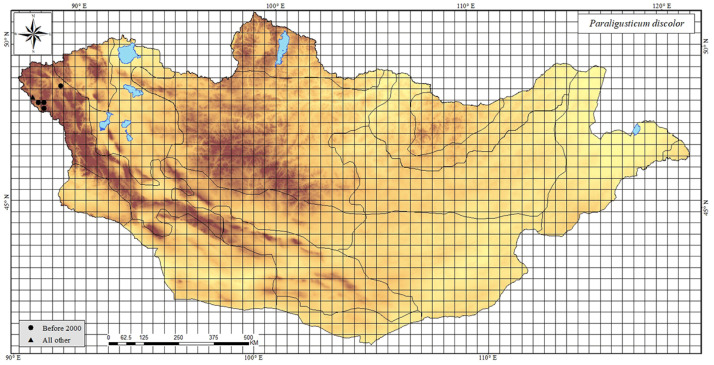
Distribution of *Paraligusticum discolor* in Mongolia.

**Figure A50:**
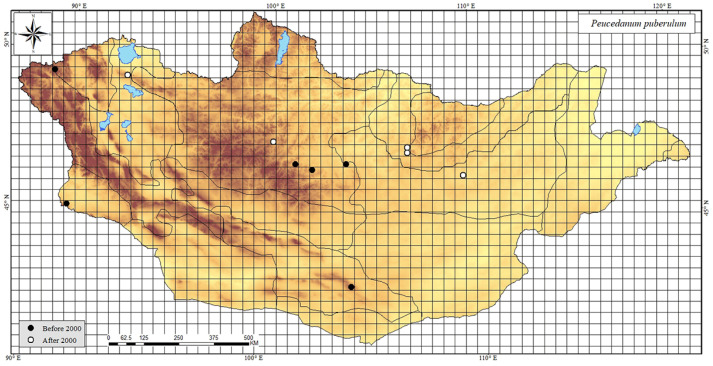
Distribution of *Peucedanum puberulum* in Mongolia.

**Figure A51:**
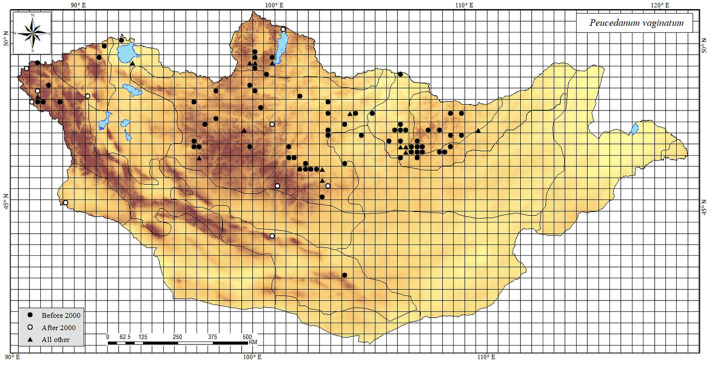
Distribution of *Peucedanum vaginatum* in Mongolia.

**Figure A52:**
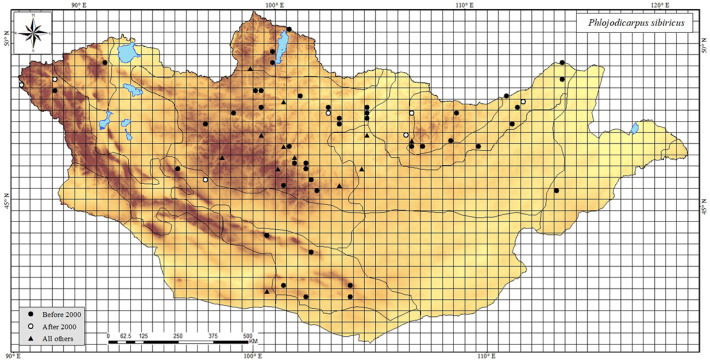
Distribution of *Phlojodicarpus sibiricus* in Mongolia.

**Figure A53:**
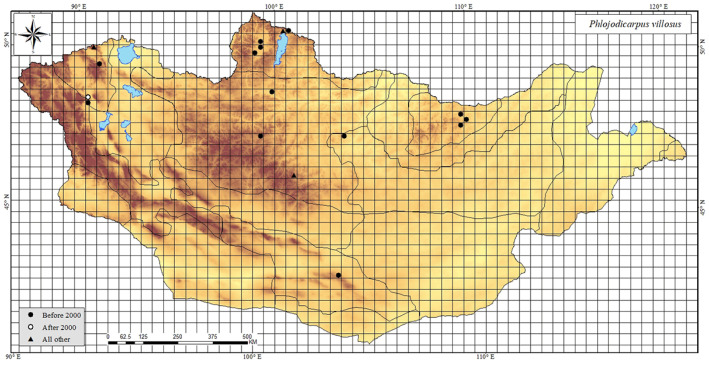
Distribution of *Phlojodicarpus villosus* in Mongolia.

**Figure A54:**
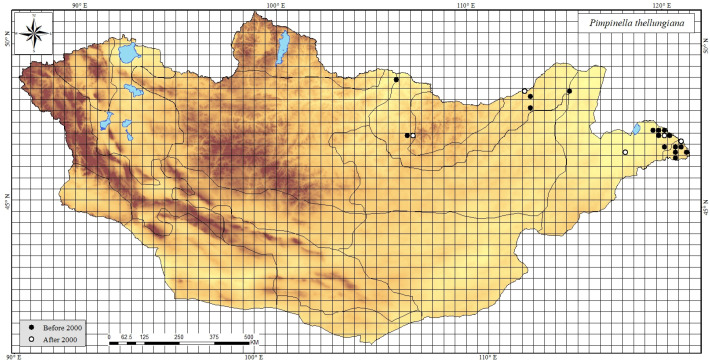
Distribution of *Pimpinella thellungiana* in Mongolia.

**Figure A55:**
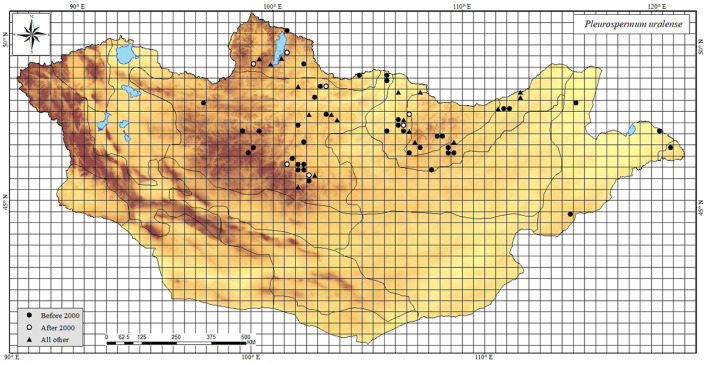
Distribution of *Pleurospermum uralense* in Mongolia.

**Figure A56:**
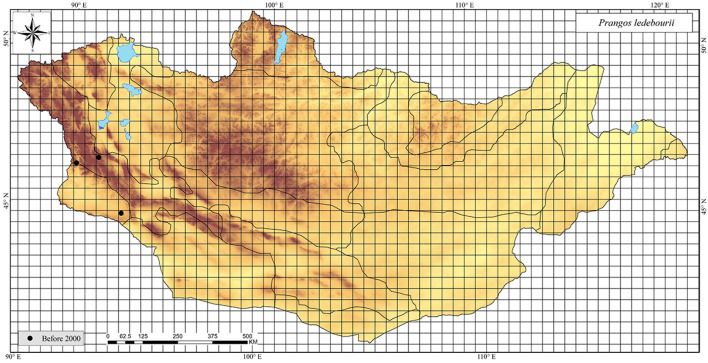
Distribution of *Prangos ledebourii* in Mongolia.

**Figure A57:**
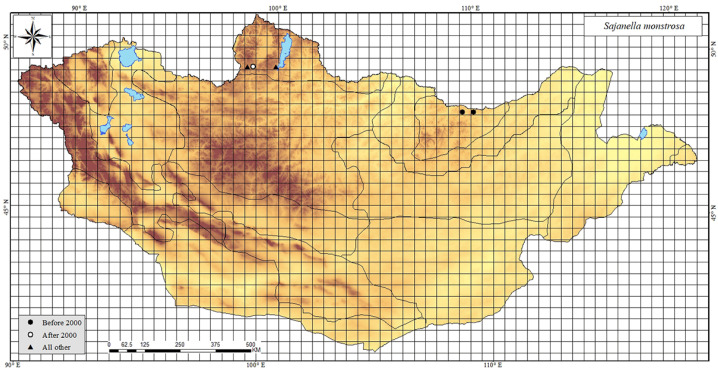
Distribution of *Sajanella monstrosa* in Mongolia.

**Figure A58:**
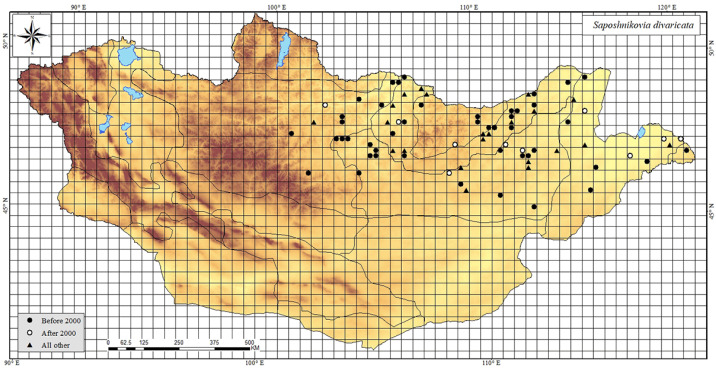
Distribution of *Saposhnikovia divaricata* in Mongolia.

**Figure A59:**
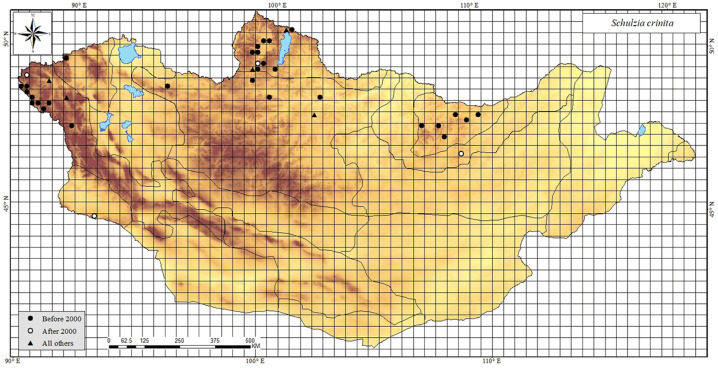
Distribution of *Schulzia crinita* in Mongolia.

**Figure A60:**
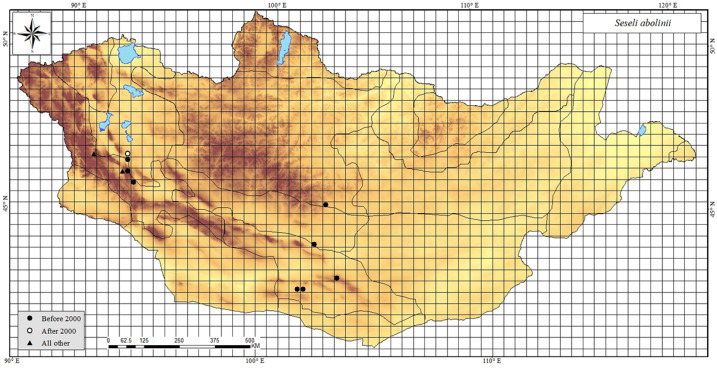
Distribution of *Seseli abolinii* in Mongolia.

**Figure A61:**
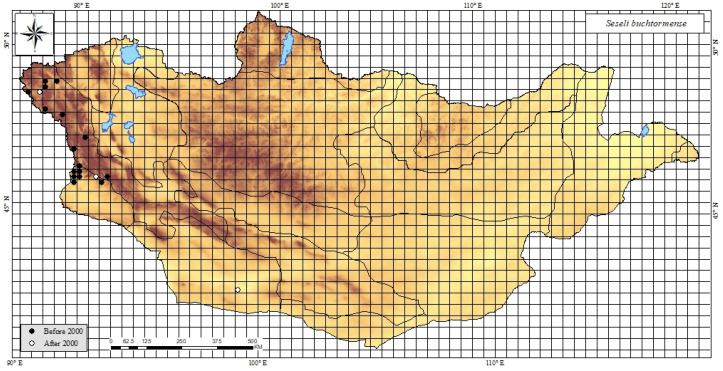
Distribution of *Seseli buchtormense* in Mongolia.

**Figure A62:**
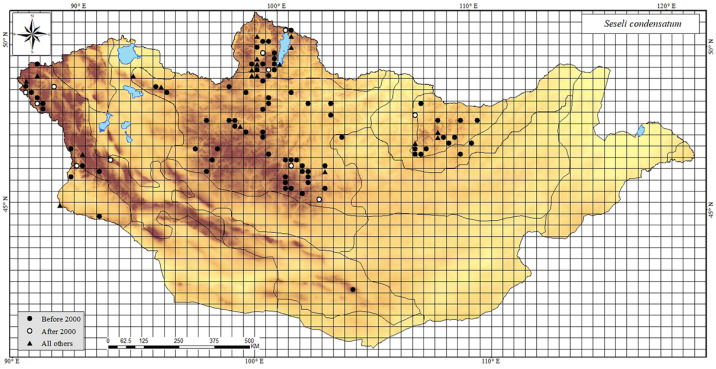
Distribution of *Seseli condensatum* in Mongolia.

**Figure A63:**
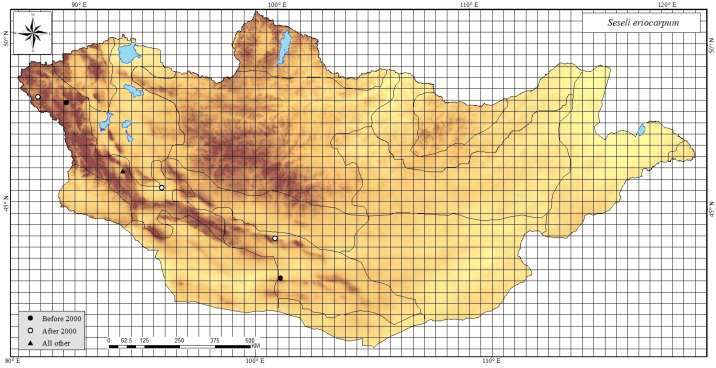
Distribution of *Seseli eriocarpum* in Mongolia.

**Figure A64:**
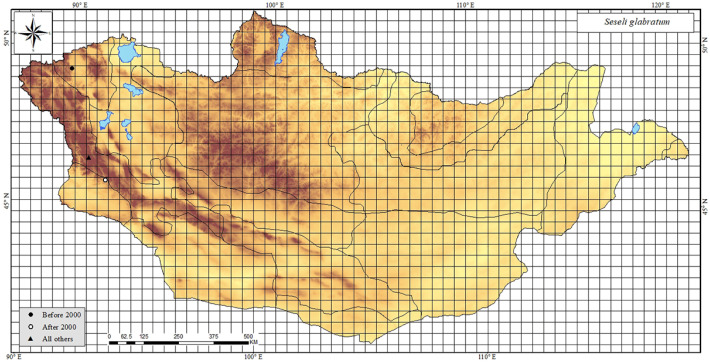
Distribution of *Seseli glabratum* in Mongolia.

**Figure A65:**
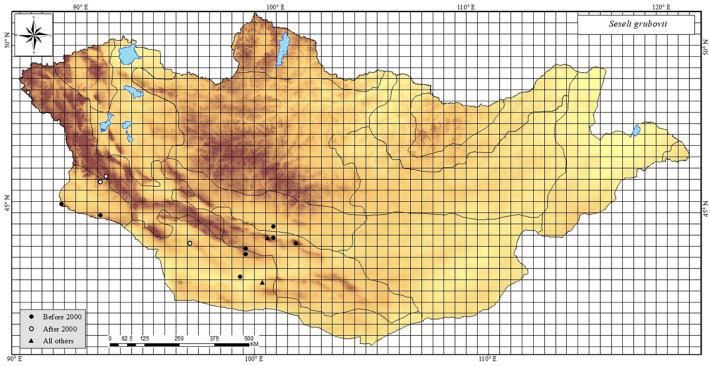
Distribution of *Seseli grubovii* in Mongolia.

**Figure A66:**
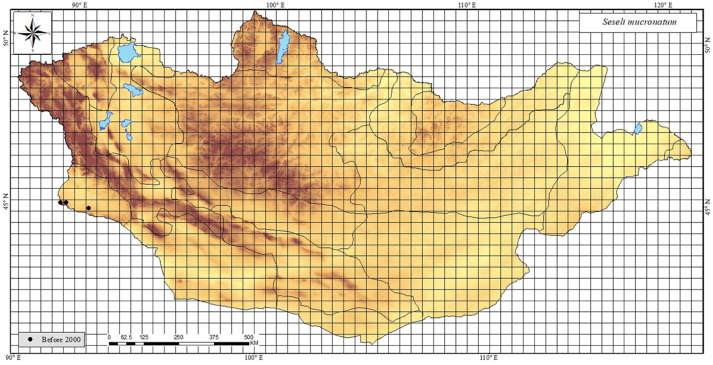
Distribution of *Seseli mucronatum* in Mongolia.

**Figure A67:**
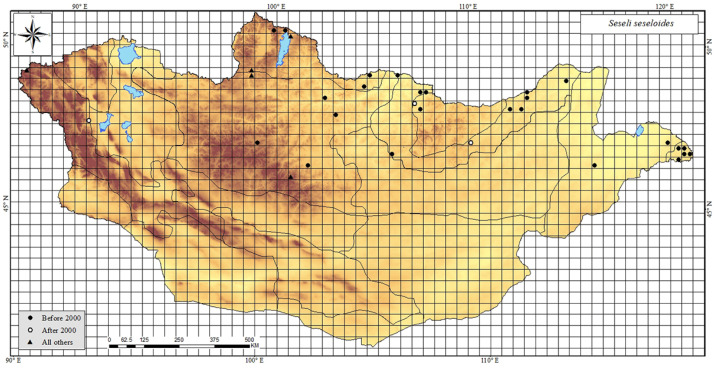
Distribution of *Seseli seseloides* in Mongolia.

**Figure A68:**
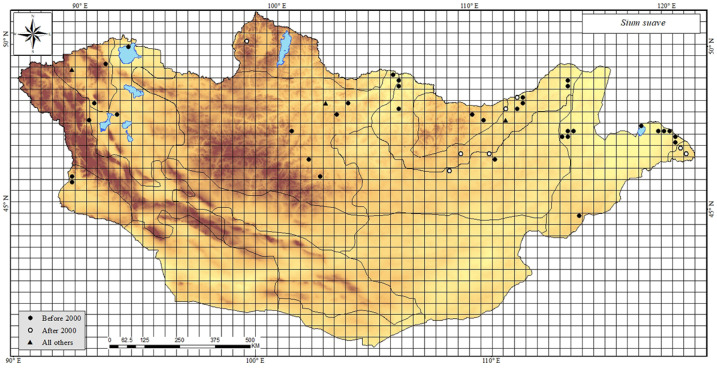
Distribution of *Sium suave* in Mongolia.

**Figure A69:**
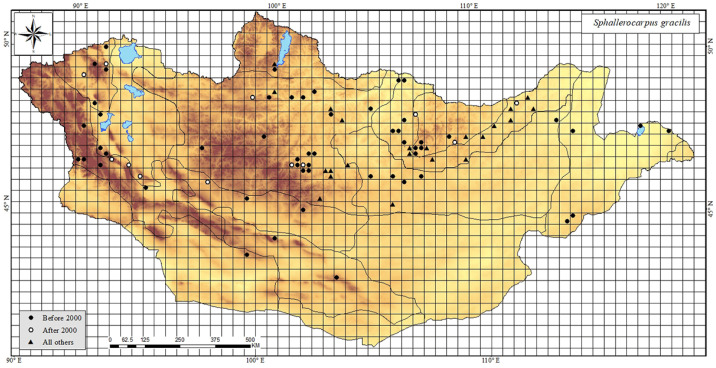
Distribution of *Sphallerocarpus gracilis* in Mongolia.

**Figure A70:**
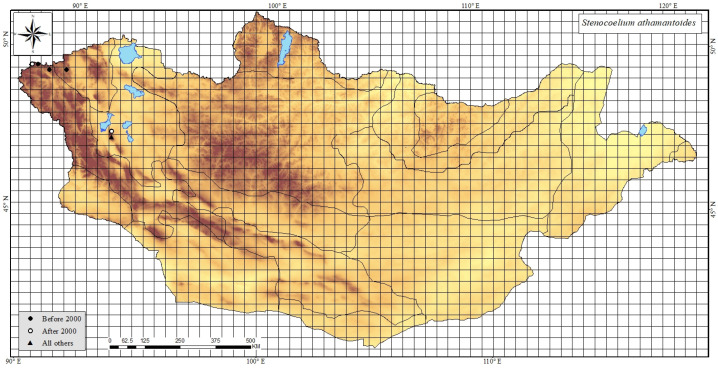
Distribution of *Stenocoelium athamantoides* in Mongolia.

## Data Availability

No new data were created or analyzed in this study. Data sharing is not applicable to this article.

## Appendix A. Annotated Checklist of the Apiaceae Family with Conservation Status and Regional Distribution

**Table A1:** Annotated checklist of the Apiaceae family with conservation status and regional distribution.

No	Accepted Taxon Name	Number of Records	EOO km^2^	AOO km^2^	Threat Category and Criteria in This Study	Previous Threat Category with Reference	Phytogeographical Regions
1	*Aegopodium alpestre* Ledeb.	128	562,142	428	LC	-	1–5, 8+, 13
2	*Angelica czernaevia* (Fisch and C.A.Mey.) Kitag.	8	2894	32	VU B1a+B2a	-	5, 9
3	*Angelica dahurica* (Hoffm.) Benth. and Hook.f.	21	302,422	68	LC	-	2–5,9
4	*Angelica saxatilis* Turcz.	1		4	CR B2a	-	2
5	*Angelica sylvestris* L.	5	153,400	16	VU B2a	-	1+, 6, 7
6	*Anthriscus sylvestris* (L.) Hoffm.	22	398,896	76	LC	-	1–5, 7, 9
7	*Archangelica decurrens* Ledeb.	69	587,817	244	LC	-	1–4, 6, 7, 14
8	*Aulacospermum anomalum* Ledeb.	8	145,059	32	VU B2a	EN [14]	6, 7
9	*Bupleurum aureum* Fisch. ex Hoffm.	7	10,118	20	EN B2a	-	7
10	*Bupleurum bicaule* Helm.	294	1,401,721	1040	LC	-	1–13, 14+
11	*Bupleurum densiflorum* Rupr.	1		4	CR B2a	-	7+, 13+, 14+
12	*Bupleurum krylovianum* Schischk. (SE)	7	48,101	24	VU B2a	-	3, 7
13	*Bupleurum multinerve* DC.	54	518,858	196	LC	-	1–7, 9, 11
14	*Bupleurum scorzonerifolium* Willd.	213	1,096,567	664	LC	-	1–6, 8, 9, 12, 13
15	*Bupleurum sibiricum* Vest.	60	413,268	204	LC	-	2–4, 8, 9
16	*Carum buriaticum* Turcz.	107	1,115,103	376	LC	-	1–6, 8, 9
17	*Carum carvi* L.	151	972,274	464	LC	-	1–10, 14
18	*Cenolophium denudatum* (Hornem.) Tutin.	11	108,196	28	VU B2a	NT [14]	2+, 4+, 7, 10, 14
19	*Cicuta virosa* L.	83	1,183,666	296	LC	-	1–15
20	*Cnidium dauricum* (Jacq.) Turcz.	74	896,555	276	LC	-	1+, 2–10
21	*Cnidium monnieri* Cusson.	9	137,823	36	VU B2a	-	2+, 4, 9
22	*Conioselinum longifolium* Turcz. (SE)	16	613,699	64	LC	-	1, 2, 4, 9, 10, 14+
23	*Conioselinum tataricum* Hoffm.	96	362,294	344	LC	-	1–4
24	*Elwendia setacea* (Schrenk) Pimenov and Kljuykov.	1		4	CR B2a	EN [14]	7
25	*Ferula bungeana* Kitag. (SE)	77	885,091	288	LC	-	8–16
26	*Ferula caspica* M.Bieb.	17	33,037	64	NT B1a+B2a	-	7, 14
27	*Ferula dissecta* Ledeb.	5	134,334	20	EN B2a	-	3, 7, 14, 15+
28	*Ferula dshaudshamyr* Korovin.	7	13,241	24	VU B1a+B2a	-	7, 14
29	*Ferula ferulioides* (Steud.) Korovin.	11	4611	32	EN B2a	EN [12]	7
30	*Ferula potaninii* Korovin.	2		8	EN B2a	-	14
31	*Ferula songarica* Pall. ex Willd.	53	257,441	204	LC	-	3, 7, 10, 14, 15+
32	*Ferulopsis hystrix* (Bunge) Pimenov. (SE)	165	1,393,906	616	LC	-	1+, 2–4, 6–15
33	*Haloselinum falcaria* (Turcz.) Pimenov. (SE)	35	528,427	136	LC	-	3, 6–8, 10, 11, 13–15
34	*Hansenia mongholica* Turcz. (SE)	9	286,325	36	VU B2a	-	1, 2
35	*Heracleum dissectum* Ledeb.	146	1,299,977	420	LC	-	1–7, 9, 13
36	*Heracleum sibiricum* L.	24	316,357	64	LC	-	1–3, 4+
37	*Kadenia salina* (Turcz.) Lavrova and V.N.Tikhom.	44	1,010,857	176	LC	-	1+, 2–4,6+, 7–11, 13
38	*Kitagawia baicalensis* (Redow.) Pimenov. (SE)	163	1,072,026	532	LC	-	1–4, 6, 8, 10, 13+
39	*Kitagawia terebinthacea* (Fisch.) Pimenov.	11	48,425	28	VU B2a	VU [14]	2, 4, 5, 9
40	*Lithosciadium kamelinii* (V.M.Vinogr.) Pimenov. (SE)	7	19,856	28	VU B1a+B2a	EN [14]	7
41	*Lithosciadium multicaule* Turcz. (SE)	33	596,884	96	LC	-	1, 3, 4, 6, 7, 13
42	*Neogaya simplex* Meisn.	102	953,174	380	LC	-	1–4, 6, 7, 13, 14
43	*Oenanthe aquatica* (L.) Poir.	1		4	CR B2a	CR [14]	10
44	*Ostericum tenuifolium* (Pall.) Y.C.Chu	94	690,102	296	LC	-	1–10, 14+
45	*Paraligusticum discolor* (Ledeb.) V.N.Tikhom.	7	2211	28	EN B2a	-	7
46	*Peucedanum puberulum* Turcz. (SE)	12	157,298	36	VU B2a	-	2, 3, 7+, 8, 13, 14+
47	*Peucedanum vaginatum* Ledeb.	130	964,839	464	LC	-	1–4, 6–8, 10+, 13, 14+
48	*Phlojodicarpus sibiricus* Koso-Pol.	74	1,201,846	256	LC	-	1–4, 6+, 7–9, 11+, 13
49	*Phlojodicarpus villosus* Turcz.	19	678,545	76	LC	-	1–3, 6, 7+, 13+
50	*Pimpinella thellungiana* H.Wolff	28	177,961	108	NT B1a+B2a	-	4, 5, 9
51	*Pleurospermum uralense* Hoffm.	106	639,543	312	LC	-	1–5, 8, 9
52	*Prangos ledebourii* Herrnst and Heyn.	3	6059	12	EN B2a	-	7, 14
53	*Sajanella monstrosa* (Willd.) Soják. (SE)	6	10,618	20	EN B2a	EN [14]	1+, 2
54	*Saposhnikovia divaricata* (Turcz.) Schischk.	129	462,209	392	LC	VU [14]	2–5, 8, 9
55	*Schulzia crinita* (Pall.) Spreng.	53	672,447	180	LC	-	1–4, 6, 7, 14+
56	*Seseli abolinii* (Korovin) Schischk.	11	172,272	44	VU B2a	-	7, 10, 11, 13
57	*Seseli buchtormense* W.D.J.Koch	26	107,933	104	LC	-	7, 14, 15+
58	*Seseli condensatum* Rchb.f.	155	956,132	508	LC	-	1–3, 4+, 7–8, 13+, 14
59	*Seseli eriocarpum* B.Fedtsch.	6	80,862	24	VU B2a	-	7, 13+
60	*Seseli glabratum* Willd.	3		8	EN B2a	-	6+, 7
61	*Seseli grubovii* V.M.Vinogr and Sanchir. (SE)	18	233,295	68	LC	-	7, 13–15
62	*Seseli mucronatum* (Schrenk) Pimenov and Sdobnina	6	453	24	EN B1a+B2a	-	14
63	*Seseli seseloides* (Fisch and C.A.Mey.) M.Hiroe.	39	764,906	144	LC	-	1–5, 7, 9
64	*Sium suave* Walter.	60	987,532	220	LC	-	1–10, 14
65	*Sphallerocarpus gracilis* Koso-Pol.	160	1,108,433	436	LC	-	1–4, 6–11, 13
66	*Stenocoelium athamantoides* Ledeb.	8	13,316	28	VU B1a+B2a	VU [14]	6, 7

*Note:* Phytogeographical regions: 1—Khuvsgul (Khu), 2—Khentii (Khe), 3—Khangai (Kha), 4—Mongolian Dauria (MD), 5—Foothills of Great Khingan (FGKh), 6—Khovd (Kho), 7—Mongolian Altai (MA), 8—Middle Khalkh (MKh), 9—Eastern Mongolia (EM), 10—Depression of Great Lakes (DGL), 11—Valley of Lakes (VL), 12—Eastern Gobi (EG), 13—Gobi Altai (GA), 14—Dzungarian Gobi (DzG), 15—Trans-Altai Gobi (TAG), 16—Alashan Gobi (AG). IUCN categories: Critically Endangered (CR), Endangered (EN), Vulnerable (VU), Near Threatened (NT), and Least Consern (LC). Subendemic areas (SE). The new locations for each region are indicated via plus symbols.
